# The Incidence, Risk Factors and Outcomes of Postoperative Acute Kidney Injury in Neurosurgical Critically Ill Patients

**DOI:** 10.1038/s41598-017-04627-3

**Published:** 2017-06-26

**Authors:** Yujun Deng, Jie Yuan, Ruibin Chi, Heng Ye, Dong Zhou, Sheng Wang, Cong Mai, Zhiqiang Nie, Lin Wang, Yiling Zhai, Lu Gao, Danqing Zhang, Linhui Hu, Yiyu Deng, Chunbo Chen

**Affiliations:** 1 Department of Critical Care Medicine, Guangdong General Hospital, Guangdong Academy of Medical Sciences, Guangzhou, 510080 Guangdong Province P.R. China; 2 Department of Critical Care Medicine, Xiaolan Hospital of Southern Medical University, Zhongshan, 528415 Guangdong P.R. China; 3 Department of Critical Care Medicine, Guangzhou Nansha Central Hospital, Nansha, 511400 Guangdong P.R. China; 4 Department of Neurosurgery, Guangdong General Hospital, Guangdong Academy of Medical Sciences, Guangzhou, Guangdong 510080 P.R. China; 5 Department of Anesthesiology, Guangdong Cardiovascular Institute and Guangdong General Hospital, Guangdong Academy of Medical Sciences, Guangzhou, Guangdong 510080 P.R. China; 6 Department of Cardiovascular Epidemiology, Cardiac Surgery, Guangdong Cardiovascular Institute, Guangdong General Hospital, Guangdong Academy of Medical Sciences, Guangzhou, Guangdong 510080 P.R. China

## Abstract

We investigated the incidence, perioperative risk factors, and outcomes of postoperative acute kidney injury (AKI) in neurosurgical critically ill patients. A prospective multicenter cohort study was conducted, enrolling adult patients who underwent neurosurgical procedure and admitted to the neurosurgical intensive care units (ICU). Postoperative AKI was diagnosed within 7 days after surgery based on the Kidney Disease Improving Global Outcomes criteria. Of 624 enrolled patients, postoperative AKI occurred in 84 patients. AKI was associated with increased rates of ICU and in-hospital mortality, postoperative renal replacement therapy, postoperative tracheotomy, and postoperative tracheal reintubation. Patients who developed AKI had higher total ICU costs, prolonged length of hospital and ICU stay, and longer duration of postoperative mechanical ventilation. Multivariate analysis identified postoperative reoperation (adjusted odds ratio [OR] 5.70 [95% CI, 1.61–20.14]), postoperative concentration of serum cystatin C (adjusted OR 4.53 [95% CI, 1.98–10.39]), use of mannitol during operation (adjusted OR 1.97 [95% CI, 1.13–3.43]), postoperative APACHE II score (adjusted OR 1.11 [95% CI, 1.06–1.16]), and intraoperative estimated blood loss (adjusted OR 1.04 [95% CI, 1.00–1.08]) as independent risk factors for postoperative AKI. Postoperative AKI in neurosurgical critically ill cohort is prevalent and associated with adverse in-hospital outcomes.

## Introduction

Postoperative acute kidney injury (AKI) is a highly prevalent and prognostically important complication in various surgical settings. Patients who developed postoperative AKI is independently associated with markedly increased morbidity, mortality^1–8^ and higher economic burden^1^. A considerable amount of publications have evaluated the incidence, determinants, and consequences of AKI in patients undergoing cardiac surgery^4, 9, 10^ or non-cardiac surgery^2, 5, 11–13^. Consequently, several preventive and treatment strategies have been developed. Nevertheless, little is known about the incidence, risk factors and outcomes of postoperative AKI in neurosurgical critically ill patients, and hence may lead to an unacceptable delay in initiating any therapy regimens.

A better knowledge of the clinical characteristics of postoperative AKI in neurosurgical critically ill cohort may help develop efficacious intervention of this complication. The aims of this study were to demonstrate the incidence of postoperative AKI in neurosurgical critically ill population, identify perioperative risk factors, and clarify the relationship between AKI and in-hospital outcomes.

## Results

### Patient’s preoperative characteristics

**Figure 1:**
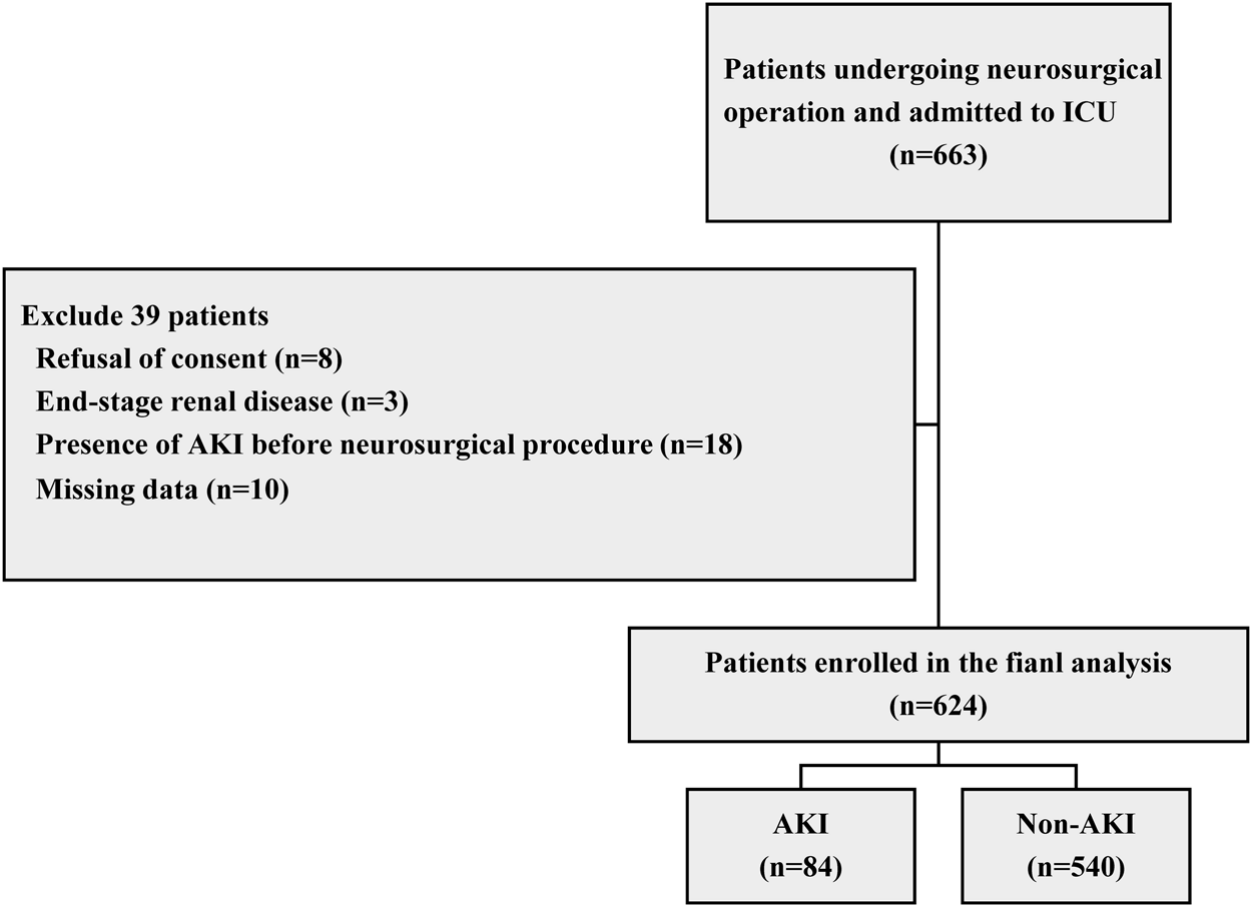
Flow chart from recruitment to outcome. Abbreviations: ICU, intensive care unit; AKI, acute kidney injury.

Of the 663 consecutive patients who were screened for inclusion in the study, 39 (5.9%) were excluded (Fig. 1). 624 patients were enrolled for analysis. Table 1 presents preoperative characteristics of patients. Overall, AKI based on the Kidney Disease Improving Global Outcomes (KDIGO) criteria occurred in 84 patients (13.5%) within the first 7 days after neurosurgical operation. Of these AKI patients, 72 patients (85.7%) were at stage 1, 8 patient (9.5%) at stage 2, and 4 patients at stage 3 (4.8%). Among the 84 AKI patients, 14 developed AKI in the first day after operation, 57 in the second day, 10 in the third day, and 3 patients beyond 3 days. Thus, 96.4% of the patients reached AKI within 3 days after operation. Preexisting chronic kidney disease (CKD) was more frequent in patients with AKI. AKI patients had higher rate of emergency surgery when compared with non-AKI patients. Moreover, patients with AKI had higher American Society of Anesthesiologists (ASA) classification. However, there are no significant differences in age, sex, body mass index (BMI), baseline serum creatinine, baseline estimated glomerular filtration rate (eGFR), and preoperative hemoglobin concentration between patients with and without AKI. Additionally, AKI patients took no more nephrotoxic drugs preoperatively than non-AKI patients. There was also no significant difference of preoperative application of radiographic contrast or mannitol between AKI and non-AKI patients. Most of the patients (73.9%) underwent operation for intracranial tumor.

**Table 1:** Preoperative Patient Characteristics by Status of AKI^a^.

Characteristics	All patients (n = 624)	Non-AKI (n = 540)	AKI (n = 84)	*P* value
Age, years	50 (40, 60)	50 (40, 59)	54 (39, 65)	0.056
Gender (male), n (%)	283 (45.4)	244 (45.2)	40 (46.4)	0.831
BMI, kg/m^2^	22.5 (20.0, 25.2)	22.3(19.9, 25.2)	22.8 (20.1, 24.6)	0.845
Preexisting clinical conditions, n (%)				
Hypertension	91 (14.6)	79 (14.6)	12 (14.3)	0.934
Diabetes mellitus	34 (5.4)	28 (5.2)	6 (7.1)	0.440
CKD	12 (1.9)	6 (1.1)	6 (7.1)	0.002
Cerebrovascular disease	32 (5.1)	27 (5.0)	5 (6.0)	0.789
Hyperlipidemia	20 (3.2)	15 (2.8)	5 (6.0)	0.171
CAD	9 (1.4)	6 (1.1)	3 (3.6)	0.108
Prior neurological surgery, n (%)	54 (8.7)	45 (8.3)	9 (10.7)	0.470
Emergency surgery, n (%)	54 (8.7)	40 (7.4)	14 (16.7)	0.005
ASA classification, n (%)				0.018
I	431 (69.1)	382 (70.7)	49 (58.3)	
II	118 (18.9)	101 (18.7)	17 (20.2)	
III	27 (4.3)	23 (4.3)	4 (4.8)	
IV	42 (6.7)	30 (5.6)	12 (14.3)	
V	6 (1.0)	4 (0.7)	2 (2.4)	
Preoperative hemoglobin, g/L	130 (119, 141)	130 (119, 141)	129 (119, 141)	0.761
Baseline serum creatinine, mg/dl	0.69 (0.59, 0.84)	0.69 (0.59, 0.84)	0.68 (0.53, 0.91)	0.704
Baseline eGFR, ml/min/1.73 m^2^	106.5 (94.8, 117.3)	106.5 (95.1, 117.3)	106.7 (87.4, 120.3)	0.488
Preoperative medication, n (%)				
Nephrotoxic drugs^b^	18 (2.9)	15 (2.8)	3 (3.6)	0.723
Radiographic contrast	26 (4.2)	22 (4.1)	4 (4.8)	0.768
Mannitol	102 (16.3)	86 (15.9)	16 (19.0)	0.472
Diagnostic group, n (%)				0.169
Spine disease	32 (5.1)	31 (5.7)	1 (1.2)	
Hydrocephalus	22 (3.5)	19 (3.5)	3 (3.6)	
Intracranial tumor	461 (73.9)	399 (73.9)	62 (73.8)	
Traumatic brain injury	16 (2.6)	12 (2.2)	4 (4.8)	
Intracranial aneurysm/AVM	27 (4.3)	22 (4.1)	5 (6.0)	
Hypertensive cerebral hemorrhage	10 (1.6)	7 (1.3)	3 (3.6)	
Others	56 (9.0)	50 (9.3)	6 (7.1)	

^a^Continuous variables were expressed as mean ± SD or median (25th percentile–75th percentile, IQR). Categorical variables were expressed as a number (%). ^**b**^Includes any of the following medications administered within 5 days before operation: nonsteroidal anti-inflammatory drug, angiotensin-converting enzyme inhibitor, angiotensin receptor blocker, immunosuppressant, aminoglycoside, vancomycin, acyclovir, amphotericin. Abbreviations: AKI, acute kidney injury; BMI, body mass index; CKD, chronic kidney disease; CAD, coronary artery disease; ASA classification, American Society of Anesthesiologists classification; eGFR, estimated glomerular filtration rate; AVM, arteriovenous malformation.

### Patient’s intraoperative characteristics

Table 2 demonstrates intraoperative parameters in this cohort. Intraoperative estimated blood loss in patients undergoing postoperative AKI was more than that in non-AKI patients. In addition, the percentage of patients who received mannitol or blood transfusion (platelets) during operation was significantly higher in AKI patients than in non-AKI patients. However, patients with AKI were not treated with more crystalloid and colloid (including hydroxyethyl starch) during operation in comparison with patients without AKI.

**Table 2:** Intraoperative Variables by Status of AKI^a^.

Variables	All patients (n = 624)	Non-AKI (n = 540)	AKI (n = 84)	*P* value
General anesthesia, n (%)	620 (99.4)	537 (99.4)	83 (98.8)	0.444
Duration of surgery, minute	290 (190, 380)	285 (185, 370)	300 (210, 410)	0.172
Estimated blood loss, per 100 ml	2.0 (1.0, 4.4)	2.0 (1.0, 4.0)	3.0 (1.5, 6.0)	0.022
Minimum MAP, mm Hg	73.3 (67.3, 78.7)	73.3 (67.7, 78.6)	72.5 (66.8, 79.8)	0.719
Radiographic contrast, n (%)	12 (1.9)	11 (2.0)	1 (1.2)	1.000
Intraoperative fluids				
Total crystalloid, per 500 ml	3.0 (2.0, 4.4)	3.0 (2.0, 4.4)	3.1 (2.0, 4.2)	0.925
Total colloid, per 500 ml	2.0 (1.0, 2.0)	2.0 (1.0, 2.0)	2.0 (1.0, 2.5)	0.077
Volume of HES, per 500 ml	1.0 (0.5, 2.0)	1.0 (0.5, 2.0)	1.0 (0.0, 2.0)	0.889
Mannitol, n (%)	124 (19.9)	97 (18.0)	27 (32.1)	0.002
RBCs, n (%)	119 (19.1)	97 (18.0)	22 (26.2)	0.074
Plasma, n (%)	62 (9.9)	53 (9.8)	9 (10.7)	0.798
Platelets, n (%)	6 (1.0)	3 (0.6)	3 (3.6)	0.035

^a^Continuous variables were expressed as mean ± SD or median (25th percentile–75th percentile, IQR). Categorical variables were expressed as a number (%). Abbreviations: AKI, acute kidney injury; MAP, mean arterial pressure; HES, hydroxyethyl starch; RBC, red blood cell.

### Patient’s postoperative characteristics

Patients who experienced AKI were likely to have lower hemoglobin levels after operation, higher concentrations of postoperative serum cystatin C (CysC) and serum creatinine (sCr) than patients without AKI (Table 3). Furthermore, they were more likely to undergo reoperation after the first neurosurgical procedure. The severity of disease scores [the postoperative Acute Physiology and Chronic Health Evaluation score (APACHE II) and Glasgow Coma Scale (GCS) score] for patients were also showed. Compared with patients without AKI, patients with AKI were more severely ill.

**Table 3:** Postoperative Variables by Status of AKI^a^.

Variables	All patients (n = 624)	Non-AKI (n = 540)	AKI (n = 84)	*P* value
APACHE II score	8 (7, 11)	8 (7, 11)	11 (8, 15)	<0.001
GCS score	15 (15, 15)	15 (15, 15)	15 (13, 15)	<0.001
Serum Cr, mg/dl	0.75 (0.63, 0.92)	0.74 (0.63, 0.91)	0.81 (0.65, 1.00)	0.037
Serum CysC, mg/L	0.73 (0.62, 0.87)	0.73 (0.61, 0.86)	0.81 (0.67, 1.03)	<0.001
Hemoglobin, g/L	114.48 ± 0.74	115.29 ± 0.80	109.27 ± 1.86	0.005
UP, ml/kg/h	2.2 (1.6, 2.9)	2.2 (1.6, 2.8)	2.4 (1.7, 3.4)	0.102
Postoperative reoperation, n (%)	12 (1.9)	6 (1.1)	6 (7.1)	0.002

^a^Continuous variables were expressed as mean ± SD or median (25th percentile–75th percentile, IQR). Categorical variables were expressed as a number (%). Abbreviations: AKI, acute kidney injury; APACHE II, Acute Physiology and Chronic Health Evaluation; GCS, Glasgow Coma Score; Cr, creatinine, CysC, Cystatin C; UP, urine production within the first 24 hours after operation; Postoperative reoperation, underwent the second neurosurgical operation within 7 days after first neurosurgical procedure.

### Multivariable analysis of risk factors that are related to postoperative AKI

Risk factors which are significantly correlated with the incidence of postoperative AKI are depicted in Table 4. The independent risk factors for postoperative AKI included estimated blood loss during operation, postoperative reoperation, use of mannitol during operation, concentration of postoperative serum CysC, and postoperative APACHE II score. Intraoperative estimated blood loss may increase the rate of AKI occurrence in an amount-dependent manner, with each 100 ml estimated blood loss increasing the odds of AKI 1.04-fold. The risk for developing AKI was 5.70 times higher in reoperation patients. Each unit increment in postoperative serum CysC was independently associated with AKI (adjusted odds ratio [OR] 4.53 [95% CI, 1.98–10.39]) after adjustment for clinical covariates. Additional, the risk for postoperative AKI was 1.97 times higher in those used mannitol during operation. We also found that higher postoperative APACHE II score could predict AKI occurrence (adjusted OR 1.11 [95% CI, 1.06–1.16]).

**Table 4:** Multivariable Logistic Regression Analysis of Factors that Are Related to Postoperative AKI in Neurosurgical Critically Ill Patients.

Variable	ORunadj	ORadj	95% CI	*P* value
Estimated blood loss during operation (per 100 ml)	1.04	1.04	1.00–1.08	0.052
Postoperative reoperation	6.85	5.70	1.61–20.14	0.007
Postoperative sCysC (per mg/L)	4.56	4.53	1.98–10.39	<0.001
Use of mannitol during operation	2.16	1.97	1.13–3.43	0.016
Postoperative APACHE II score	1.13	1.11	1.06–1.16	<0.001

Abbreviations: AKI, acute kidney injury; ORunadj, odds ratio unadjusted; ORadj, odds ratio adjusted; CI, confidence interval; Postoperative reoperation, reoperation within 7 days after the first neurosurgical operation; APACHE II, Acute Physiology and Chronic Health Evaluation; sCysC, serum Cystatin C.

### In-hospital outcomes and postoperative AKI

In the present study, AKI occurrence was associated with higher rates of postoperative renal replacement therapy (RRT), intensive care unit (ICU) mortality, in-hospital mortality, postoperative tracheotomy, and tracheal reintubation (Table 5). Moreover, patients with AKI had higher total ICU costs, prolonged hospital and ICU length of stay, and longer duration of postoperative mechanical ventilation.

**Table 5:** Postoperative Clinical Outcomes by Status of AKI^a^.

Outcomes	All patients (n = 624)	Non-AKI (n = 540)	AKI (n = 84)	*P* value
Duration of mechanical ventilation, hours	3 (1, 6)	3 (1, 5)	5 (3, 20)	<0.001
Reintubation, n (%)	19 (3.0)	10 (1.9)	9 (10.7)	<0.001
Tracheotomy, n (%)	19 (3.0)	11 (2.0)	8 (9.5)	0.002
RRT, n (%)	2 (0.3)	0 (0)	2 (2.4)	0.018
ICU mortality, n (%)	13 (2.1)	2 (0.4)	11 (13.1)	<0.001
Hospital mortality, n (%)	15 (2.4)	2 (0.4)	13 (15.5)	<0.001
ICU length of stay, days	1 (1, 2)	1 (1, 2)	2 (1, 3)	<0.001
Hospital length of stay, days	14 (10, 18)	13 (10, 18)	15 (11, 23)	0.029
Total ICU costs, CNY	8888 (6959, 13470)	8549 (6894, 12758)	11658 (8126, 23985)	<0.001

^a^Continuous variables were expressed as mean ± SD or median (25th percentile–75th percentile, IQR); Categorical variables were expressed as a number (%). Abbreviations: AKI, acute kidney injury; RRT, renal replacement therapy ICU, intensive care unit; CNY, Chinese yuan.

## Discussion

In the multi-center prospective study, AKI occurred frequently and was associated with adverse in-hospital outcomes in neurosurgical critically ill population. The independent risk factors of postoperative AKI occurrence included intraoperative estimated blood loss, postoperative reoperation, use of mannitol during operation, concentration of postoperative serum CysC, and postoperative APACHE II score.

Concomitant with social and economic development, the number of neurosurgical operations increased worldwide^14, 15^ and great progress has been made in neurosurgery. Although a retrospective study reported the incidence of AKI after craniotomy in a large cohort was 9.9%^16^. Prospective study about the incidence, determinants and consequences of postoperative AKI in entire neurosurgical critically ill patients is scarce. Therefore, we conducted a prospective study to assess the incidence, risk factors and outcomes of AKI occurrence in such a relatively large multicenter neurosurgical cohort.

Our study showed that the rate of AKI occurrence was up to 13.5% in patients undergoing neurological surgery within the first 7 days. The incidence of AKI in our study is similar to those reported among several non-cardiac surgery cohorts, which varied from 7.5% to 24%^2, 5, 12, 13^, but lower than those in cardiac surgery^4, 17^. Although several studies had reported the incidence of AKI in traumatic brain injury cohorts, ranging from 8% to 25%^18–21^, the postoperative AKI incidence of entire neurosurgical critically ill cohort has not been well described. Our investigation provided more evidences regarding incidence of postoperative AKI in such specific population.

The risk factors of postoperative AKI varied in different clinical settings, and 5 aforementioned risk factors were identified in this neurosurgical cohort. In previous studies^3, 22^, it has been reported that AKI was significantly related to the estimated blood loss during operation. In the present study, intraoperative estimated blood loss was the independent risk factor of postoperative AKI occurrence. On one hand, excessive bleeding may lead to hemodynamic compromise and hemoglobin reduction. Anemia, caused by significant hemoglobin reduction, can reduce renal oxygen delivery, promote oxidative stress, and impair hemostasis^4, 23^, which would contribute to AKI occurrence. On the other hand, excessive bleeding may necessitate RBC (red blood cell) transfusions. Owing to progressive structural and functional changes of preserved RBCs during storage, transfused stored RBCs may weaken tissue oxygen delivery, promote a proinflammatory state, and exacerbate tissue oxidative stress^24^, which are also associated with AKI. Intraoperative hypovolemia which is related to intraoperative blood loss or inadequate fluids therapy may contribute to AKI development. However, continuous monitoring volume status has not been routinely conducted during neurosurgical procedure. Therefore, the value of volume status monitoring in high-risk group need to be confirmed with respect to postoperative AKI in neurosurgical cohort.

In our study, reoperation is another determinant for postoperative AKI. Our result is consistent with previous studies^4, 25^, which reported that reoperation was independently associated with AKI. Although the mechanisms of AKI caused by reoperation have not been fully clarified, the logical assumption is that they involve exacerbation of many of the factors, such as hemodynamic compromise, bleeding and operative trauma, which are related to AKI occurrence.

CysC is a low molecular weight protein (13 kDa)^26^, and considered as a reliable, functional marker for renal function. Recent studies indicated that CysC is an early predictor of AKI^27, 28^ and also has reasonable discrimination for adverse outcomes^29^. A previous study found^30^ that the increase of serum CysC preceded that of creatinine for 1–2 days in acute kidney failure detection. In the present study, the concentration of postoperative serum CysC at ICU admission, instead of serum creatinine, was an independent risk factor of postoperative AKI. Although the postoperative level of serum creatinine was associated with increased risk of AKI on univariate analysis, this relationship did not persist with multivariable adjustment. It is worthy of note that both serum CysC and creatinine are clinically available biomarkers in China and abroad, our result implies serum CysC may be a better renal biomarker for AKI prediction than serum creatinine in such population.

As many previous studies have reported the close association between mannitol and kidney injury^31–33^, we evaluated the relation between this drug and AKI. Our study demonstrated that use of mannitol during operation, instead of preoperative use, was an independent risk factor for postoperative AKI with a 1.97-fold increase in the risk-adjusted odds. Although the mechanisms that the use of mannitol is close associated with AKI occurrence are not completely elucidated, its possible reason is that use of mannitol might lead to swelling of proximal tubular cells and vacuolization. Undoubtedly, this would result in deterioration of kidney function^31^. In addition to the toxic impact on kidney, the association of AKI with intraoperative use of mannitol may also be attributed to illness severity such as high intracranial pressure or cerebral edema. Generally, intraoperative use of mannitol signifies more serious neuropathophysiological insults which are associated with high risk of AKI^18, 34^.

Both APACHE II and GCS scoring systems are commonly used to evaluate the severity of critically ill patients^35^. However, in our study, APACHE II score but not GCS score, is independently associated with postoperative AKI. As APACHE II score is a physiologically based system containing 12 physiological parameters, it is a useful prediction tool of hospital consequences, such as mortality and AKI, in critically ill patients^36^. Moreover, APACHE II score system includes GCS score, and thus, pathophysiological changes predicting in an organism after systemic insult could be illustrated comprehensively and systematically by the APACHE II scoring system. Therefore, this scoring system is thought to be superior to GCS for prediction of adverse outcome^37^. In the present study, APACHE II score, instead of GCS score, is an independent predictor of postoperative AKI, probably owing to the same reason.

The commonly prescribed medications that predispose to renal impairment were also analyzed in our study. Although it is well known that nephrotoxic drugs (nonsteroidal anti-inflammatory drug, angiotensin-converting enzyme inhibitor, angiotensin receptor blocker, immunosuppressant, aminoglycoside, vancomycin, acyclovir, or amphotericin) have kidney impairment effect, the use of these drugs was not associated with AKI in the present study. The reason that we could not get statistically significant conclusions may be attributed to a small numbers of patients using these drugs in our cohort.

It is increasingly evident that even relatively modest kidney injury is independently associated with an increased risk for morbidity and mortality^38, 39^. In comparison with non-AKI patients, we found that AKI patients was significantly associated with in-hospital and ICU mortality, and other adverse outcomes, which was consistent with previous studies^2, 4, 5, 9, 11^. Although lacking effective therapy regimens at present, evaluating modifiable risk factors for AKI may contribute to developing novel strategies for preventing postoperative AKI in neurosurgical critically ill patients. Hence we attempted to find out modifiable predict factors predisposing to AKI. Of note, all aforementioned identified risk factors in our study are potential modifiable. Further intervention study should be performed to prove effectiveness regarding these modifiable risk factors before translating our research into clinical application.

There are several limitations that should be addressed in the present study. Similar to all observational research, the variables chosen as possible risk factors were based on available literature and investigator’s hypotheses, but the effects of residual or unmeasured confounders on the observed associations between the risk factors and AKI cannot be ruled out. Furthermore, the follow-up duration was limited to the period of hospitalization, and thus, post-discharge outcomes could not be accounted for in the present analysis. Additionally, due to a small number of events, statistical power was limited to detect the impact of AKI on mortality, which is a hard endpoint. This may limit the ability to interpret our data on mortality. Last but not least, almost 74% of the patients in our cohort have had surgery for intracranial tumor. Therefore, the generalization of our conclusion to all neurosurgical critically ill patients may be limited. However, this disproportionate size of the intracranial tumor group reflected the real situation of three ICUs in the present study.

In conclusion, the present study showed that postoperative AKI based on KDIGO criteria occurred in 13.5% of neurosurgical critically ill patients. Independent risk factors for postoperative AKI were intraoperative estimated blood loss, postoperative reoperation, postoperative concentration of serum CysC, use of mannitol during operation, and postoperative APACHE II score. Postoperative AKI was closely related to adverse in-hospital outcomes. Therapies aimed at mitigating these risk factors may offer protection against this complication. Thus, this study could have significant clinical implications for neurosurgical critically ill patients at risk for postoperative AKI.

## Methods

### Study Design and Participants

The prospective observational study was conducted in the neurosurgical ICUs of three tertiary care hospitals in China. Patients aged 18 years or older who underwent neurosurgical procedure and admitted to the neurosurgical ICUs between January 2015 and April 2016 were included. The exclusion criteria included refusal of consent, preexisting end-stage renal disease or renal dysfunction requiring RRT before operation, preexisting renal transplantation, preexisting nephrectomy, presence of AKI before neurosurgical procedure, or missing admission data. The primary outcome was the occurrence of AKI within 7 days after neurosurgical operation. The protocol was in accordance with STROBE (Strengthening the Reporting of Observational Studies in Epidemiology) guidelines^40^. All experiments were performed in accordance with the approved protocols, guidelines, and regulations. This study was approved by the Ethics Committee of the Guangdong General Hospital, as well as the committees of other two participating centers (the Ethics Committee of Xiaolan Hospital of Southern Medical University and the Ethics Committee of Guangzhou Nansha Central Hospital), and all patients (or appropriate surrogates for patients unable to consent) provided written informed consent.

### Data Collection

Each patient’s clinical data were prospectively collected from electronic hospital and laboratory databases. The following variables were recorded: age, gender, BMI, preexisting clinical conditions [hypertension, diabetes mellitus, CKD, cerebrovascular disease, hyperlipidemia, and coronary artery disease (CAD)], prior neurological surgery, emergency surgery, ASA classification, preoperative use of nephrotoxic drugs (nonsteroidal anti-inflammatory drug, angiotensin-converting enzyme inhibitor, angiotensin receptor blocker, immunosuppressant, aminoglycoside, vancomycin, acyclovir, or amphotericin), preoperative administration of radiographic contrast or mannitol, and diagnostic group (spine disease, hydrocephalus, intracranial tumor, traumatic brain injury, intracranial aneurysm/arteriovenous malformation, hypertensive cerebral hemorrhage, or others). Laboratory values were obtained, including level of preoperative hemoglobin, baseline serum creatinine, and concentration of postoperative serum creatinine, hemoglobin, and CysC at ICU admission. Blood samples were measured at the central laboratory of the Guangdong General Hospital using a standard protocol. Serum creatinine and hemoglobin were measured before operation, and then measured after operation at ICU admission, and thereafter at least once a day as a part of routine clinical care during ICU hospitalization. Postoperative serum CysC was measured only once at ICU admission. We also recorded the hourly urine output of each patient from enrollment to ICU discharge. The baseline eGFR was calculated by the CKD-Epidemiology Collaboration Equation^41^. The APACHE II score^42^ and the GCS^43^ score were used to evaluate disease severity. The postoperative APACHE II and GCS scores were assessed immediately after patients had recovered from anesthetic. Postoperative reoperation within 7 days after first neurosurgical procedure was recorded.

Surgical data including duration of surgery, intraoperative estimated blood loss, lowest mean arterial pressure (MAP; i.e. lowest MAP for at least 5 continuous minutes) during anesthesia, amount and type of intraoperative fluids administered (crystalloid and colloid), intraoperative use of mannitol and transfusions (red blood cells, platelets, and plasma) were recorded. Artificial colloid solutions used during the study period consisted of the hydroxyethyl starch and gelatin.

Outcome variables were also recorded, including duration of postoperative mechanical ventilation, incidence of postoperative tracheal reintubation, tracheotomy and RRT, total ICU costs, ICU and in-hospital mortality, and length of stay in hospital and ICU.

### Definitions

AKI was defined based on the KDIGO criteria^44^ for AKI within one week after surgery as any of the following: increase in sCr by ≥0.3 mg/dl (≥ 26.5 µmol/l) within 48 hours, or increase in sCr to ≥1.5 times baseline within one week, or urine output <0.5 ml/kg/h for 6 hours. AKI is staged according to the following KDIGO criteria. Stage 1: increase of sCr to 1.5–1.9 times from baseline, or ≥0.3 mg/dl (≥26.5 µmol/l) increase of sCr, or urine output <0.5 ml/kg/h for 6–12 h. Stage 2: increase of sCr to 2.0–2.9 times from baseline or urine output <0.5 ml/kg/h for ≥12 h. Stage 3: 3 times increase of sCr from baseline or ≥4.0 mg/dl (≥353.6 µmol/l) increase of sCr or initiation of RRT, or urine output <0.3 ml/kg/h for ≥24 h or anuria for ≥12 h.

A baseline creatinine was determined by using the following rules ranked in descending order of preference as previously described:^45^ (1) the most recent pre-ICU value between 30 and 365 days before ICU admission (n = 54); (2) a stable pre-ICU value >365 days for patients aged <40 years, (stable defined as within 15% of the lowest ICU measurement) before ICU admission (n = 1); (3) pre-ICU value >365 days before ICU admission and less than the initial serum creatinine on ICU admission (n = 4); (4) a pre-ICU value (between 3 and 39 days before ICU admission) less than or equal to the initial on-admission serum creatinine to ICU and not obviously in AKI (n = 352); (5) the lowest of initial on-admission serum creatinine to ICU (n = 150), the last ICU value (n = 50), or the minimum value at follow-up to 365 days (n = 13).

### Statistical Methods

As reported previously described by Harrell, Vittinghoff, Steyerberg^46^, events per variable (EPV) >10 was an important issue for estimation of multivariable regression coefficients. To avoid biased estimation of regression Coefficients, EPV = 15 would be required of interest in our final outcome model. Thus, to fit a model with 5 covariates, we would require approximately 75 outcome events. We calculated the sample size based on an estimated AKI incidence of 13%, which we determined in a chart review of 100 patients undergoing neurosurgical operation (unpublished). Therefore, a sample size of 578 cases was required. Considering a possible dropout rate of 10%, we would need approximately 636 cases.

Continuous variables were expressed as medians with interquartile range or mean ± SD. Categorical variables were expressed as number (percentage). The non-normally distributed continuous variables were compared by Wilcoxon rank-sum test. To compare the categorical variables, Chi-square or Fisher’s exact test was used.

Univariate and multivariate logistic regression was used to evaluate the relationship between postoperative AKI and perioperative risk factors. The clinical perioperative variables with *P* < 0.10 in univariate analysis were included in multivariate analysis. Logistic multivariate forward stepwise (likelihood ratio) regression was then used to determine the most efficient predictors of AKI. Results are presented as ORs with 95% confidence intervals (CI). All the tests were two-tailed, and *P* < 0.05 was considered as statistical significance. SPSS version 13.0 were used.
